# Supplementary material for: ELK1/MTOR/S6K1 Pathway Contributes to Acquired Resistance to Gefitinib in Non-Small Cell Lung Cancer
Source: Int J Mol Sci. 2024 Feb 17;25(4):2382. doi: 10.3390/ijms25042382 (PMC10888698; doi:10.3390/ijms25042382)
Supplement: Supplementary file 1 [file ijms-25-02382-s001.zip › ijms-2786564-supplementary.pdf]

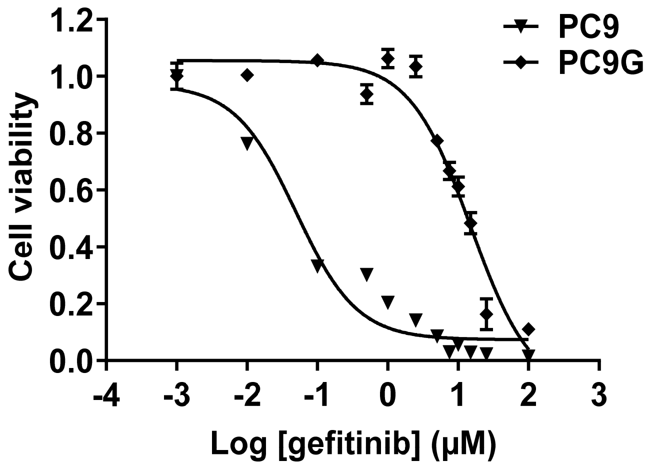

Supplemental Figure S1. PC9G is resistant to gefitinib compared to PC9 cells. The 72h IC_{50} of gefitinib in PC9 and PC9G cells was measured using an MTT assay.
